# Effect of Calebin-A on Critical Genes Related to NAFLD: A Protein-Protein Interaction Network and Molecular Docking Study

**DOI:** 10.2174/0113892029280454240214072212

**Published:** 2024-02-22

**Authors:** Ali Mahmoudi, Mohammad Mahdi Hajihasani, Muhammed Majeed, Tannaz Jamialahmadi, Amirhossein Sahebkar

**Affiliations:** 1 Student Research Committee, Faculty of Medicine, Mashhad University of Medical Sciences, Mashhad, Iran;; 2 Department of Medical Biotechnology and Nanotechnology, Faculty of Medicine, Mashhad University of Medical Sciences, Mashhad, Iran;; 3 Department of Pharmaceutical Control, School of Pharmacy, Mashhad University of Medical Sciences, Mashhad, Iran;; 4 Department of Chemistry, Sabinsa Corporation, 20 Lake Drive, East Windsor, NJ, 08520, USA;; 5 *Medical Toxicology Research Center, Mashhad University of Medical Sciences, Mashhad, Iran; *; 6 Department of Medical Biotechnology, Biotechnology Research Center, Pharmaceutical Technology Institute, Mashhad University of Medical Sciences, Mashhad, Iran;; 7 Applied Biomedical Research Center, Mashhad University of Medical Sciences, Mashhad, Iran

**Keywords:** Calebin-A, functional enrichment analysis, GEO, molecular docking, NAFLD, prediction databases, PPI network

## Abstract

**Background:**

Calebin-A is a minor phytoconstituent of turmeric known for its activity against inflammation, oxidative stress, cancerous, and metabolic disorders like Non-alcoholic fatty liver disease(NAFLD). Based on bioinformatic tools. Subsequently, the details of the interaction of critical proteins with Calebin-A were investigated using the molecular docking technique.

**Methods:**

We first probed the intersection of genes/ proteins between NAFLD and Calebin-A through online databases. Besides, we performed an enrichment analysis using the ClueGO plugin to investigate signaling pathways and gene ontology. Next, we evaluate the possible interaction of Calebin-A with significant hub proteins involved in NAFLD through a molecular docking study.

**Results:**

We identified 87 intersection genes Calebin-A targets associated with NAFLD. PPI network analysis introduced 10 hub genes (TP53, TNF, STAT3, HSP90AA1, PTGS2, HDAC6, ABCB1, CCT2, NR1I2, and GUSB). In KEGG enrichment, most were associated with Sphingolipid, vascular endothelial growth factor A (VEGFA), C-type lectin receptor, and mitogen-activated protein kinase (MAPK) signaling pathways. The biological processes described in 87 intersection genes are mostly concerned with regulating the apoptotic process, cytokine production, and intracellular signal transduction. Molecular docking results also directed that Calebin-A had a high affinity to bind hub proteins linked to NAFLD.

**Conclusion:**

Here, we showed that Calebin-A, through its effect on several critical genes/ proteins and pathways, might repress the progression of NAFLD.

## 1 INTRODUCTION

A prominent cause of chronic liver disease globally is non-alcoholic fatty liver disease (NAFLD), which influences a considerable proportion of the community in many parts of the world [1]. Hepatic steatosis, the major symptom of NAFLD, occurs when no other reasons for secondary liver fat deposition (such as excessive alcohol use) can be found. Non-alcoholic steatohepatitis (NASH) is a severe form of NAFLD that might progress into fibrosis and cirrhosis [2, 3]. Steatosis is evident in NAFLD without any signs of inflammation. Still, when coupled with lobular inflammation and apoptosis, it can lead to non-alcoholic steatohepatitis (NASH) and subsequent ramifications such as fibrosis and cirrhosis [4, 5]. The incidence of NAFLD has grown quickly in Western nations, with a global prevalence of 25%. NAFLD is linked to metabolic diseases such as central obesity, hypertension, dyslipidemia, chronic abnormalities, and hyperglycemia [6]. The gold standard for diagnosing NAFLD is histological assessment using a liver biopsy. NAFLD is diagnosed based on hepatic steatosis, and its severity varies according to the presence of lobular inflammation, ballooning, and fibrosis [6]. The growing prevalence of NAFLD worldwide, coupled with its risk of progressing to more advanced liver disease states, represents a major public health challenge. Furthermore, the lack of any pharmacological agent approved for NAFLD highlights the urgent need for novel treatment options to address this disorder.

Calebin-A was originally isolated from *Curcuma longa* [7] and then discovered in *Curcuma caesia* [8]. Various evidence has revealed the value of these plants in modern medicine as prophylactic agents against inflammation [9], oxidative stress [10], and cancer [11-13]. This secondary phytochemical indicates safe properties even at a high dose [14]. Calebin-A is an aglycone, glucuronidated metabolite that has a serum half-life of approximately 1-3 hours and largely non-renal excretion [10].

Calebin-A was found to repress adipogenesis through suppression of adipocyte differentiation at an early stage and decreasing several key modulators such as peroxisome proliferator-activated receptor γ (PPARγ), fatty acid synthase (FAS), and CCAAT/enhancer-binding protein (C/EBP) α/β in adipocytes. Calebin-A was discovered to cause lipolysis at 20 µM and simultaneously facilitate AMP-activated protein kinase (AMPK) activity. Moreover, Calebin-A could protect against metabolic syndrome and decrease hepatic steatosis in mice fed a high-fat diet (HFD) [15], along with weight reduction and blood glucose control (15). In a separate study, Calebin-A was found to exert anti-obesity effects by regulating thermogenesis and gut microbiota and enhancing intestine commensal bacteria like *Butyricoccus* and *Akkermansia* [16]. Permanent inflammatory processes are the root of the majority of illnesses. Calebin-A can impact several pro-inflammatory signaling pathways [17].

In the last decade, numerous bioinformatic tools have appeared to help scientists speed up target identification and drug discovery. This field is comprised of a combination of computer science and molecular biology. These technologies offer a wide variety of information, including genomics, transcriptomics, and proteomics, as well as epigenomics, pharmacogenomics, metagenomics, and metabolomics [18]. The most common usage of those *in silico* studies included high-throughput transcriptomics analysis and protein-protein interaction (PPI) networks to find critical targets related to particular diseases [19, 20]. Also, virtual screening and molecular docking are time- and cost-effective compared to the conventional deconvolution and investigation approach to determining the relationship between drugs and diseases [21-25]. Molecular docking is a computer-based technique that simulates the interaction of small molecules to macromolecular targets' structures and evaluates whether they could complement the binding site [26, 27].

While some previous studies have investigated the biological effects and therapeutic potential of Calebin-A, its mechanisms of action in NAFLD and specific protein targets remain unclear from an *in silico* perspective. A few studies have used high-throughput techniques to characterize differentially expressed genes in NAFLD patients and animal models. However, an integrated computational analysis linking Calebin-A, NAFLD targets, and their molecular interactions has not been reported.

In this study, we aimed to 1) identify potential protein targets of Calebin-A associated with NAFLD pathogenesis using *in silico* databases and PPI network analysis and 2) explore the molecular interactions between Calebin-A and relevant protein targets through docking studies. We hypothesize that Calebin-A modulates NAFLD by binding to and regulating key protein targets involved in hepatic lipid metabolism and inflammation. Elucidating the mechanism of Calebin-A action may aid the development of new therapeutic strategies for NAFLD. A flowchart is shown in Fig. (**1**) to depict the study's design visually.

## 2 MATERIALS AND METHODS

### 2.1 Calebin-A and Target Search

We first searched several important prediction databases like www.swisstargetprediction, https://prediction.charite.de/, http://targetnet.scbdd.com/, http://gdbtools.unibe.ch:8080/PPB/browser.html, to find the possible targets of Calebin-A. These databases have specific algorithms that could predict possible targets for Calebin-A based on the 2D/3D Structure of Calebin-A that imported data in all the databases, usually in SMILES format of the small molecules [28-31].

### 2.2 Exploring NAFLD-gene Associations

#### 2.2.1 Data Source

The expression profile GSE135251 based on GPL18573 Illumina NextSeq 500 platform was acquired from the Omnibus database (GEO). The GEO database freely and openly shares vast gene expression and other omics datasets internationally to further the molecular study of biological systems through distributed archives of high-throughput transcriptional profiles [32]. The data included 206 patients with different stages of NAFLD and 10 healthy people. We used this database to achieve an overexpressed gene set in the NAFLD.

#### 2.2.2 Identification of Differentially Expressed Genes

The GSE raw count matrix was downloaded from the GEO database. The count matrix was exported to R (version 4.2.1), and the METADATA related to the GSE number was obtained by the GEOquery package. We used the “limma” and “edgeR” packages for normalization and differential expression analysis. For normalization, DGEList and calcNormFactors methods were applied. A specific size factor for each sample was deliberated, and each gene count was divided by this size factor. NAFLD and Control were set as the independent variables for significance testing. After normalization, the differential gene expression was calculated by an exact Test based on Benjamini and Hochberg (False discovery rate). Furthermore, |logFC| >0 with adjusted *P*-value < 0.05 was considered as the cut-off for the output data differentially expressed genes (DEGs). Moreover, the samples based on the DEGs heatmap and volcano plot were drawn with the ggplot2 package for visualizing and grouping.

#### 2.2.3 Assay of the Intersection of Essential Proteins of NAFLD and Calebin A

The Venn diagram evaluated the intersection of all achieved targets from prediction databases with overexpressed genes achieved in GSE135251 (https://bioinfogp.cnb.csic.es/tools/venny/).

#### 2.2.4 Protein-protein Interaction (PPI) Network and MCODE Analysis

Based on the above methodology, the Protein-Protein interaction (PPI) network was constructed from intersection targets of two achieved databases. In this case, first, we used the STRING website (https://string-db.org/) to find the interaction among the genes/proteins. STRING is a comprehensive and integrative source that contains physical and functional protein-protein interactions based on *in silico* knowledge or experimental validation [33]. A medium confidence score >0.4 was and species limitation to the “Homo sapiens” considered. Subsequently, analysis of the PPI network was accomplished based on important centralities like Degree (as topological algorithms) and Betweenness and Closeness (as shortest paths) [34]. Moreover, the clustering was performed using the Molecular Complex Detection (MCODE) algorithm [35]. All the analyses were done using Cytoscape version 3.9.1 software with NetworkAnalyzer (version 4.4.8) and MCODE (version 2.0.2) plugins.

### 2.5 Molecular Docking

#### 2.5.1 Target Proteins and Ligand Preparation

The structure of Calebin-A in a single structure document (SDF) format was obtained from the PubChem database (https://pubchem.ncbi.nlm.nih.gov/). PubChem is an open chemistry database known as an important chemical information resource that comprises small and large molecule structures, identifiers, biological activities, and chemical and physical properties [36]. We explored the potential direct interaction of essential hub proteins related to NAFLD with Calebin-A by Molecular docking. The energy of the molecules was minimized and then transformed to PDBQT (Protein Data Bank, Partial Charge (Q), and Atom Type (T)) format to use Vina for the docking process. AutoDock Vina is a freely available computational tool for exploring possible binding conformations between macromolecules and ligand molecules through *in-silico* docking simulations [37]. The 3D structure of proteins (in PDB format) was taken from the RCSB PDB web server (http://www.pdb.org/). The RCSB PDB is a protein 3D structural database achieved with various methods like nuclear magnetic resonance (NMR) and X-ray crystallography [38].

#### 2.5.2 Molecular Docking Process

We first primed the structure of selected proteins by UCSF Chimera software (version 1.8.1), and then, the operation of docking was performed against Clebin-A in PyRx software with 500 exhaustiveness. We employed UCSF Chimera software to facilitate the preparation of ligands for molecular docking simulations. This involved utilizing the software's capabilities to add hydrogen atoms, assign partial charges, optimize structures, and address any structural issues or inconsistencies that may arise [39].

PyRx is software for docking a set of small molecules against macromolecules in one run [40]. The result of molecular docking is reported by binding energy (ΔGbind). Finally, the H-bind interactions were analyzed using Discovery Studio Visualizer 4.5.

#### 2.5.3 KEGG and Gene Ontology Enrichment Analysis

KEGG and gene ontology (Biological process, molecular functions, and cellular component) were estimated with the ClueGO+CluePedia version 1.5.9 program. ClueGO is a Cytoscape plugin for investigating the interrelationships of gene sets to achieve their function [41]. KEGG contains a collection of databases associated with genomes, diseases, biological pathways, chemical substances, and drugs [42].

#### 2.5.4 ADME Estimation

We evaluate the absorption, distribution, metabolism, and excretion (ADME) of Calebin-A to investigate their drug-likeness potential using http://www.swissadme.ch.

## 3 RESULTS

**Fig. (2):**
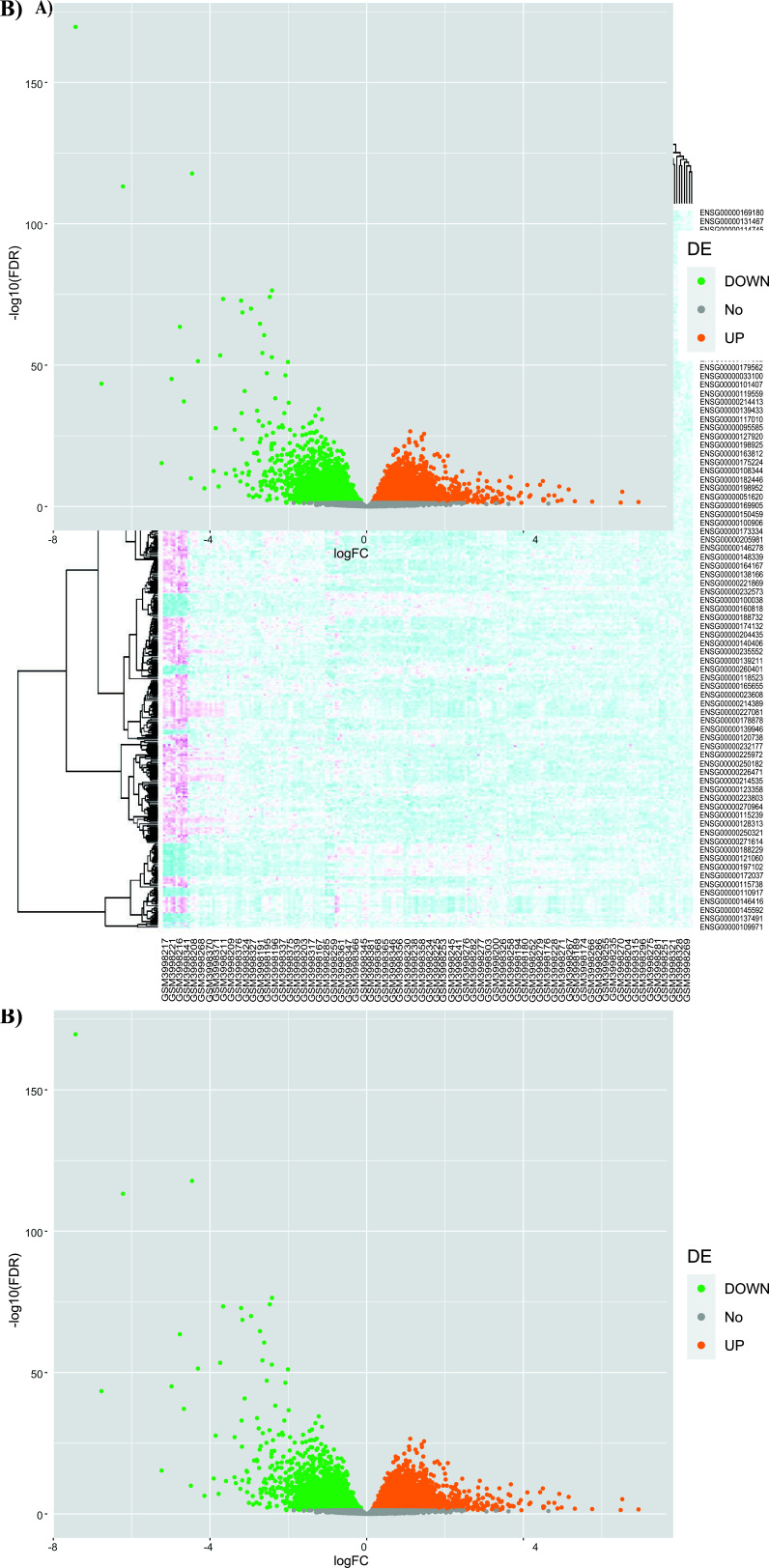
Heatmap (**A**) and volcano map (**B**) of the DEGs in GSE135251.

The investigation of the possible targets for Calebin-A in the prediction databases was reported in Table **1**. The results found 317 unique targets for Calebin-A. Conversely, the analysis of GSE135251 included 4852 up-regulated and 4042 down-regulated. The clustering of genes among the samples was indicated in the Heatmap (Fig. **2A**). Moreover, differential gene expression (Fold-change), along with significant (downregulate (green) and upregulate (orange)), is visualized in Fig. (**2B**).

**Fig. (3):**
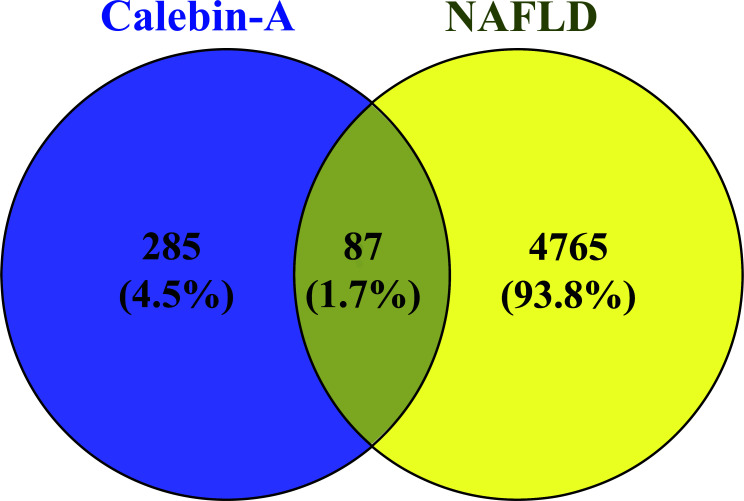
Intersection analysis of calebin-A-related targets and NAFLD-related genes using venn diagram.

Next, in Venn diagram analysis among two datasets (Calebin-A-related targets *VS* NAFLD-related genes), we observed 87 intersected genes (Fig. **3**).

**Fig. (4):**
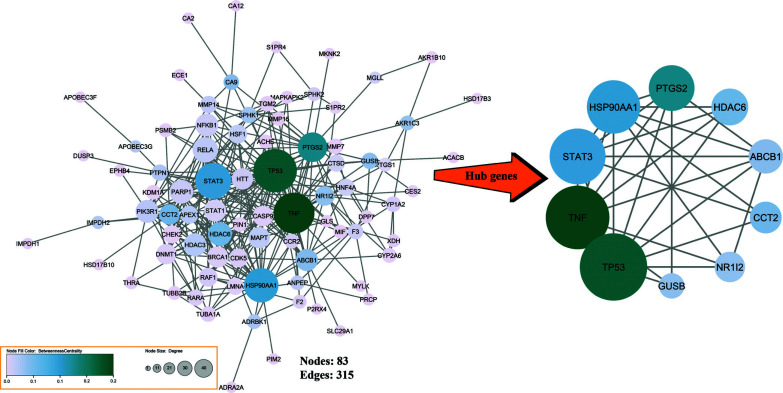
Illustration of the PPI network with 83 nodes and 315 edges and principal centralities identified in our study. The size of nodes clarifies the degree, and the intensity of node color clarifies betweenness.

The PPI network that was created contained 83 nodes and 315 edges. The analysis of the main centralities showed that Tumor Protein p53 (TP53), Tumor Necrosis Factor (TNF), Signal transducer and activator of transcription 3 (STAT3), Heat Shock Protein 90 Alpha Family Class A Member 1 (HSP90AA1), Prostaglandin-Endoperoxide Synthase 2 (PTGS2), Histone deacetylase 6 (HDAC6), ATP-binding cassette subfamily B member 1 (ABCB1), Chaperonin Containing TCP1 Subunit 2 (CCT2), nuclear receptor subfamily 1 (NR1I2), and glucuronidase beta (GUSB) are the Hub genes that have the most impact on the PPI network (Fig. **4**, Table **2**).

**Fig. (5):**
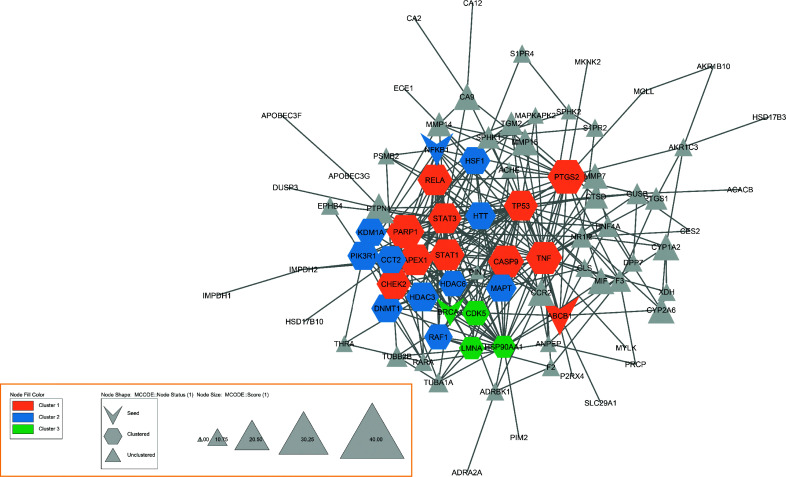
The three clusters were derived from MCODE analysis and were illustrated with different colors. The seed of each cluster is indicated with a V shape.

In addition, the MCODE analysis identified three modular clusters in the PPI network. The highest score cluster (MCODE 1) comprises 11 nodes and 40 edges with 8.00 scores and seed: ABCB1. MCODE2 was set up with 11 nodes, 22 edges, and a score of 4.40, seed: HDAC3. MCODE3 was identified with a score of 3.33, seed BRCA1, node of 4, and edge of 5 (Fig. **5**).

**Fig. (6):**
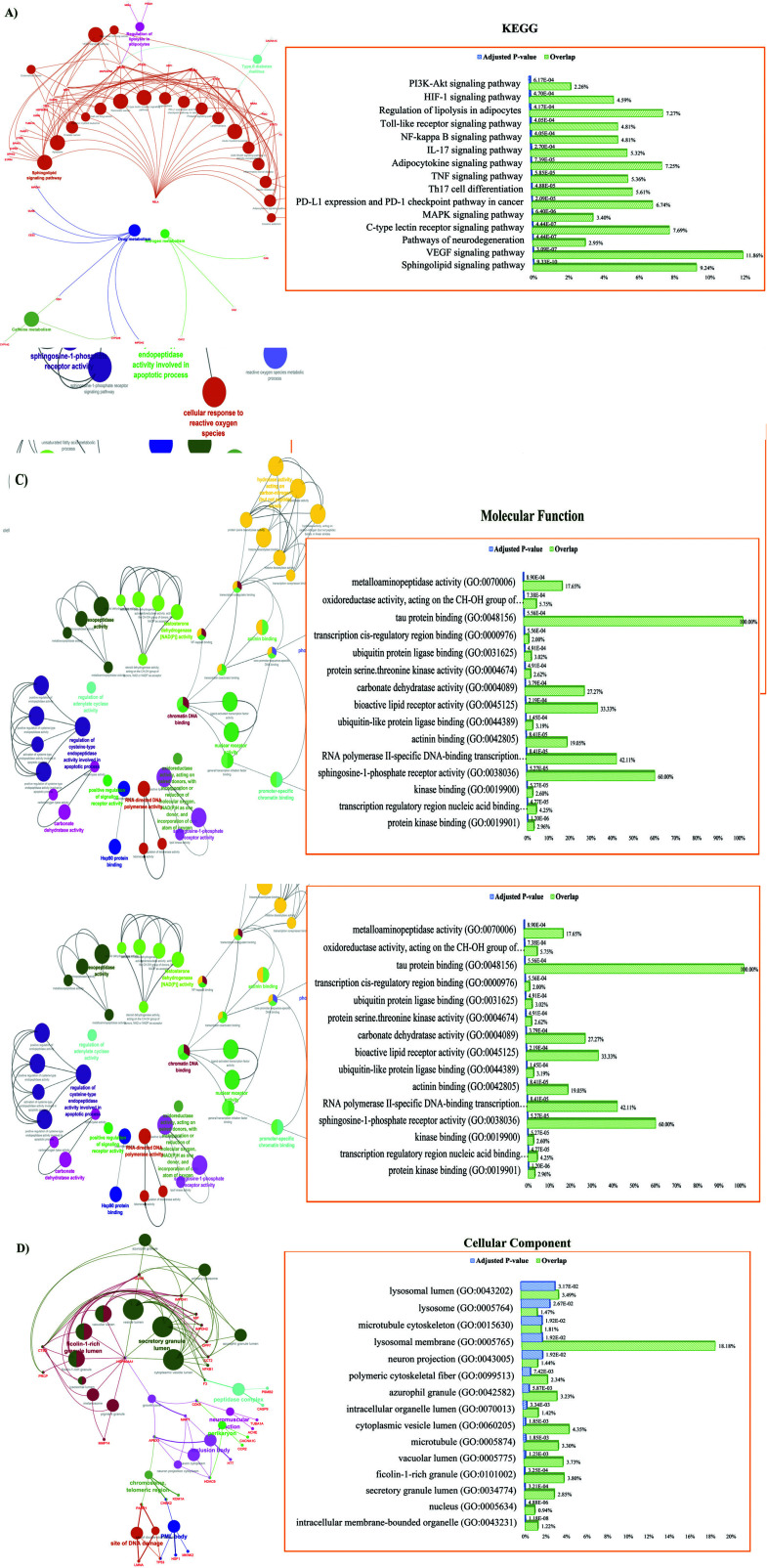
Enrichment analysis for signaling pathways and gene ontology of 87 intersection protein/ genes using ClueGO. (**A**) Fifteen highest adjusted *p*-value and signaling pathway (KEGG) (**B**) Biological process (**C**) Molecular function (**D**) Cellular component.

The highest adjusted *P*-value pathways in the KEGG enrichment of 87 shared genes/ proteins (intersection of overexpressed genes related to NAFLD ∩ Calebin-A targets) were relevant with Sphingolipid signaling pathway with 9.24% associated genes percent and *P*-value: 9.33E-10, Vascular endothelial growth factor A (VEGFA) signaling pathway with 11.86% associated genes percent and *P*-value: 3.09E-07, C- type lectin receptor signaling pathway with 7.69% associated genes percent and *P*-value: 7.69%, and Mitogen-Activated Protein Kinase (MAPK) signaling pathway with 3.40% associated genes percent and *P*-value: 6.40E-06 (Fig. **6A**).

Functional enrichment analysis at the biological process level proposed regulation of apoptotic process with 2.56% associated genes percent and *P*-value: 6.36E-07, positive regulation of cytokine production with 3.88% associated genes percent and *P*-value: 2.02E-06, positive regulation of intracellular signal transduction with 2.75% associated genes percent and *P*-value: 7.69E-06 (Fig. **6B**). Besides, under molecular function, the analysis revealed that 87 intersection proteins/ genes were mainly applied in protein kinase binding with 2.96%associated genes percent and *P*-value: 1.20E-06, transcription regulatory region nucleic acid binding with 4.25% associated genes percent and *P*-value: 4.77E-05, kinase binding with 2.60% associated genes percent and *P*-value: 5.27E-05, and sphingosine-1-phosphate receptor activity with 60.00% associated genes percent and *P*-value: 5.27E-05 (Fig. **6C**). Additionally, an intracellular membrane-bounded organelle with 1.22% associated genes percent and *P*-value: 1.18E-08, a nucleus with 0.94% associated genes percent and *P*-value: 4.88E-06, secretory granule lumen with 2.85% associated genes percent and *P*-value: 3.21E-04, and ficolin-1-rich granule with 3.80% associated genes percent and *P*-value: 3.25E-04 are discovered under cellular components (Fig. **6D**).

### 3.1 Molecular Docking Analysis

The 3D structure of Calebin-A (SDF format) obtained from PubChem (CID: 637429) is indicated in Fig. (**7**). The specification of the 3D structure of TP53, TNF, STAT3, HSP90AA1, and PTGS2 achieved from the PDB database is reported in Table **3**.

### 3.2 Pharmacokinetics and Physicochemical Properties of Calebin-A

**Fig. (9):**
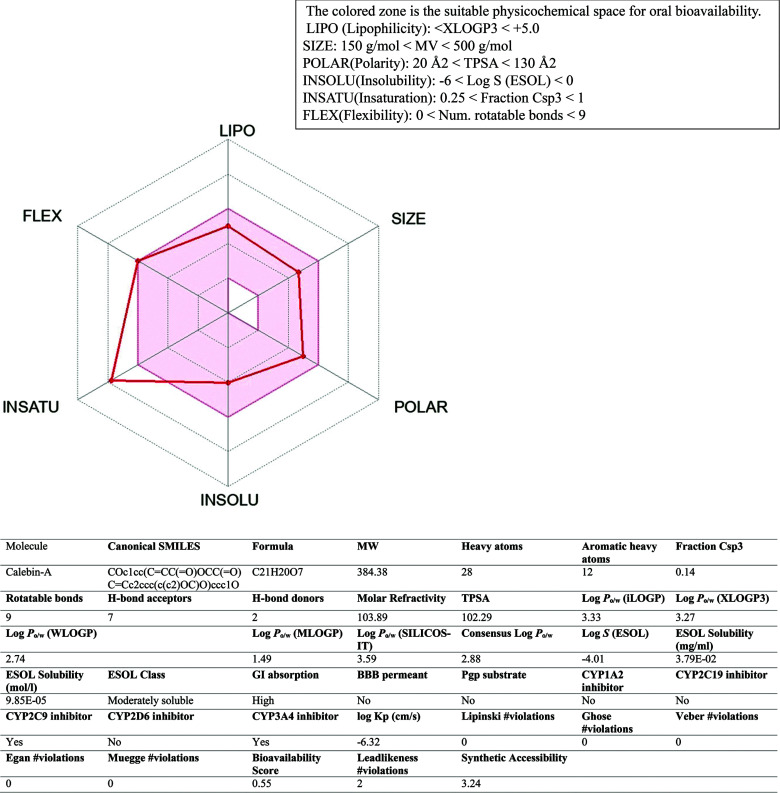
ADME properties of calebin-A by SwissADME.

The physicochemical and pharmacokinetics properties of the Calebin-A were predicted using SwissADME, and the achieved outcomes are exhibited in Fig. (**8**). Calebin-A prediction was observed to confirm Lipinski’s rule of five, Ghose, Veber, and Egan. The bioavailability score was 55%, and had good gastrointestinal (GI) absorption. Calebin-A showed non-inhibitory for several cytochrome P450 isoenzymes like CYP1A2, CYP2D6, and CYP2C19. Calebin-A, as reported by the SwissADME website, displayed good drug-likeness properties (Fig. **9**).

The docking result of Calebin-A with TNF, STAT3, TP53, PTGS2, and HSP90AA1 was reported in Table **4**, and their interactions were illustrated in Fig. (**8**). The results indicate that Calebin-A interacts with five essential hub proteins based on Binding Energy and H-bond observed. Calebin-A has a great binding with PTGS2 through five H-bonds and a binding affinity equal to -9.3 kcal/mol. It also indicated suitable interaction with STAT3 three H-bonds and -7.1 kcal/mol Binding Energy.

## 4 DISCUSSION

With the rising incidence of diabetes and obesity globally, the adverse effects of NAFLD are becoming a serious public health concern. NAFLD is one of the most common chronic liver diseases that can rapidly progress to other disorders, such as hepatocellular cancer. Calebin-A is a newly identified natural product that has structural similarity to curcumin, the bioactive pigment of turmeric [43-52]. Here, by using *in silico* tools, we showed the possible effect of Calebin-A on several critical genes involved in NAFLD. We demonstrated that Calebin-A could potentially target TP53, TNF, STAT3, HSP90AA1, PTGS2, HDAC6, ABCB1, CCT2, NR1I2, and GUSB, which are overexpressed in patients with NAFLD. We also showed that Calebin-A could impact several lipids metabolisms- and inflammation-related pathways and biological processes that participate in the progression of NAFLD. However, the most influenced pathway was the sphingolipid signaling pathway with SPHK2, SPHK1, PIK3R1, S1PR2, RAF1, TNF, CTSD, TP53, S1PR4, RELA, and NFKB1, VEGF signaling pathway with CASP9, SPHK2, SPHK1, MAPKAPK2, PIK3R1, RAF1, and PTGS2, C-type lectin receptor signaling pathway STAT1, MAPKAPK2, PIK3R1, RAF1, PTGS2, TNF, RELA, and NFKB1, and MAPK signaling pathway with DUSP3, MAPKAPK2, MKNK2, MAPT, CACNA1C, RAF1, TNF, TP53, RELA, and NFKB1. According to previous studies, these pathways were suggested to be involved in NAFLD.

Sphingolipids are linked to the development of hepatic insulin resistance [53, 54]. It has been shown that sphingosine-1-phosphate/ ceramide balance controls cell biology linked to hepatic damage stimulated by HFD [55].

An *in-vitro* study indicated that VEGFA could trigger human hepatic stellate cell (HSC) LX2 to take on a fibrogenic phenotype through VEGF-VEGFR signaling in a fatty acid media. Moreover, in the NAFLD-HCC group, a positive link between hepatic fibrosis and VEGFA was observed [56].

MAPK signaling pathway controls the nuclear factor E2-related factor 2 (Nrf2) and nuclear factor kappa B (NF-κB), which are implicated in liver and metabolic illnesses [57, 58]. In a mice model of diet-induced obesity, activation of MAPK signaling led to fat accumulation, inflammation, and the generation of reactive oxygen species (ROS) [59]. The advancement of NAFLD is accelerated by MAPK signaling, which also causes lipid buildup and inflammatory reactions [60, 61]. A promising approach for treating NAFLD/NASH is inhibiting the NF-B and MAPK cascades, reversing hepatic steatosis, inflammation, and aberrant lipid metabolism [62].

Calebin-A could potentially target TP53, TNF, STAT3, HSP90AA1, and PTGS2, which are implicated in NAFLD. In the gene expression (logFC: 0.91, *P*-Value: 9.04E-03) and PPI network (highest betweenness score: 0.23, Degree: 37, and Closeness score: 0.60) results, TNF was discovered as an essential hub gene involved in NAFLD that could be affected by Calebin-A. Docking analysis also indicated that Calebin-A could bind to the TNF-α (-6.5 kcal/mol) with two H-bonds, possibly suppressing TNF-α expression. TNF-α affects several processes connected to the pathogenic mechanisms of several human illnesses, including cellular activation and proliferation, inflammation, immune response, and cell death [63]. Calebin-A is crucial in controlling inflammatory responses and is connected to the pathogenesis of various inflammatory and autoimmune diseases [64]. Previous studies have shown that TNF-α is necessary for initiating NAFLD and its development into NASH by upregulating key molecules involved in inflammatory cytokines, lipid metabolism, and liver fibrosis [65]. Due to its role as the primary regulator of inflammatory cytokines, TNF-α has recently become recognized as a therapeutic target for several illnesses, including NAFLD. The development of NAFLD has been associated with activating pro-inflammatory cytokines, including TNF, in hepatocytes and adipose tissue [66]. Liver fat accumulation causes activation signals similar to the NF-kB *via* the upstream activation of IKK. TNF-α and other significant pro-inflammatory mediators are produced as a result of this activation, which induces the activation of Kupffer cells [67, 68]. TNF-α was highly up-regulated in the hepatocytes generated from mice fed with HFD. Earlier research also suggested that a high-carbohydrate diet (NAFLD-model) enhanced the amount of TNF-α in mouse liver [69, 70]. According to a randomized clinical trial, TNF-α was also considerably overexpressed in NAFLD patients [71]. Patients with steatohepatitis had greater blood TNF-α levels than healthy individuals with simple steatosis [72-74]. Moreover, the fibrotic stages were linked to the elevation of TNF and TNF-receptor-1 expression in the liver of patients with steatohepatitis [75]. In a preclinical study, Tomita *et al.* also confirmed that TNFR-deficient or TNF-deficient animal models of genetic or diet-induced NAFLD had better insulin sensitivity and less pronounced steatosis and fibrosis in the liver [76]. Calebin-A has been evaluated for its anti-inflammatory activity in several studies. Calebin-A could prevent IκBα degradation, nuclear translocation of p65-NF-κB, and NF-κB binding to DNA, thus preventing TNF-α induced canonical NF-κB activation [77, 78]. Other studies have reported that Calebin-A could block TNF-β like TNF-α [79-81].

In the pathogenesis of liver illnesses, the signal transducer and activator of transcription-3 (STAT3) plays a crucial role [4]. STAT3 belongs to the Janus kinase (JAK)/STAT pathway and is crucial in causing liver injury [82]. Deficiencies in STAT3 DNA-binding were reported to be mediated by increasing the expression of Pias3 in liver fibrosis [83]. STAT3 proteins were detected in high concentrations in the nucleus of proliferating biliary epithelial cells and hepatocytes from the liver of patients with cirrhosis [84]. STX-0119, a STAT3 dimerization inhibitor, was suggested to slow the progression of liver fibrosis by preventing the activation of hepatic stellate cells [85].

According to numerous investigations, STAT3 activity is also a survival signal that guards against lipotoxicity, whereas blocking hepatic STAT3 activation with other drugs reduces NAFLD-induced liver fibrosis [82]. In the NAFLD population, progressive fibrosis was linked to phosphorylation of STAT3, which is associated with an elevated risk for hepatocellular carcinoma (HCC) [86]. Recent research has shown that PNPLA3-mediated susceptibility to NAFLD was reduced by decreasing IL-6/STAT3 activity but increasing it in wild-type liver cells, promoting NAFLD onset. It was concluded that this function is due to bearing the rs738409 SNP that led to enhancing NF-κB activity, which is the cause of the heightened IL-6/STAT3 [87]. Calebin-A might regulate inflammatory pathways such as JAK/STAT3. The Janus kinases (JAKs), non-receptor cytoplasmic tyrosine kinases, are the primary promoters of STAT activation [17]. Several studies have suggested that curcumin may control the STAT3 signaling pathway. Most of these studies focus on STAT3's function in malignancies and the role of curcumin in suppressing them. Curcumin was shown to reduce STAT3 phosphorylation and block STAT3-mediated signaling [88]. In a previous study using an animal model of colitis, it was discovered that curcumin treatment drastically reduced STAT3 dimer DNA-binding activity and phospho-STAT3 activity [89]. A recent study found that STAT3 expression levels decreased in MDA-MB-231 cells following curcumin treatment [90]. By directly interfering with STAT3-mediated carcinogenesis, curcumin can abort atypical STAT3 activity [91]. However, no study has investigated the effect of Calebin-A on STAT3. In our PPI network analysis, we discovered STAT3 as a key protein interaction with a degree score of 31, a betweenness score of 0.58, and a closeness of 0.53, suggesting its importance in NAFLD. Calebin-A could modulate STAT3 through three H-bonds with a binding affinity energy of -7.1 kcal/mol.

TP53 is the genome's security factor, primarily as a tumor suppressor. It modulates a broad range of signaling pathways that inhibit oncogenic transformation [92]. p53 is thought to be a key player in the pathophysiology of NAFLD [93-98]. TP53 was discovered to be overexpressed in the livers of many NAFLD-plagued mouse models [98]. In normal sterol circumstances, TP53 directly and SREBP2 independently suppress the production of SQLE (squalene epoxidase), the first oxygenation enzyme and a rate-limiting step in cholesterol synthesis [99]. In mice given a HFD, TP53 activation was associated with hepatocyte apoptosis [96]. Moreover, in liver biopsy samples of human patients, there was a significant association between the degree of steatosis and p53 expression [97]. In a mouse model of NAFLD, the TP53 inhibitor pifithrin-p-nitro (PFT) caused attenuating steatosis, oxidative stress, and apoptosis [95]. According to these results, TP53 activation may be a widespread metabolic process that plays a significant role in the pathogenesis of fatty liver, regardless of the underlying cause, and that promotes apoptosis and oxidative stress and the development of harmful hepatic abnormalities like insulin resistance and steatosis. In one study, Calebin-A was shown to induce G2/M cycle arrest in human colon cancer cells by lowering the levels of the proteins cdc25A, cyclin A, cyclin B, and cdc2, and raising the levels of CDKIs like P21and TP53. Calebin-A also elevated levels of ROS, prompted a DNA damage response, and enhanced H2AX, chk2, and chk1 phosphorylation. Calebin-A administered intraperitoneally also dramatically reduced tumor diameter and volume [100]. Our obtained data displays that TP53 is one of the critical genes based on PPI network analysis (degree score: 40, betweenness score: 0.20, and closeness score: 0.63), which could be a possible target for NALFD. Calebin-A could strongly interact with TP53 by 6 H-bonds (-6.2 kcal/mol).

The HSP90AA1 is responsible for encoding heat shock protein 90α, which is triggered by stress and controls inflammation *via* several mechanisms. Previous research demonstrated that Hsp90α levels were higher in the serum of individuals with NAFLD, and there was a significant link between serum Hsp90 levels and steatohepatitis activity degree [101]. HSP90AA1 has been identified as one of the hub proteins in a PPI network analysis of GSE109836 [102]. In another *in silico* investigation of two datasets (GSE74656 and GSE62232), it was indicated that HSP90AA1 is a new pathogenic gene in the progression of NAFLD to HCC. The expression of HSP90AA1 was reported to be considerably decreased [103] in the liver of patients with alcoholic fatty liver disease (87). The level of HSP90AA1 could be repressed by curcumin administration [104]. However, there are no reports on the effect of Calebin-A on HSP90AA1. We observed HSP90AA1 as an effective hub gene in the PPI network, and the interaction with Calebin-A was through one H-bond (-6.9 kcal/mol).

The PTGS2 gene encodes an enzyme commonly known as cyclooxygenase two (COX-2) in the human body. The activation of PTSG2 has been linked to the etiology of several liver disorders, such as NAFLD, by enhancing hepatocyte lipid accumulation. Activation of PTSG2 after the establishment of type 2 diabetes and metabolic syndrome might have aggravating effects on the advancement of NASH [105]. PTGS2 is primarily associated with the role of gut flora in the onset and progression of NAFLD [106]. Calebin-A has been detected as a direct and non-selective inhibitor of PTSG1 and PTSG2 in several independent studies [10, 107]. As PTSG2 is one of the several NF-κB-dependent gene final products that play a significant role in many inflammatory processes, it is a main target for many traditional anti-inflammatory medicines [108]. PTGS2, discovered as an essential hub gene in DEGs and PPI network analyses, showed a strong interaction with Calebin-A (three H-bonds and a high affinity of -9.3 kcal/mol).

Throughout the early stages of drug discovery and development, computational prediction of bioavailability and drug-likeness qualities continues to be a crucial criterion in exploring drug candidates [43]. Physiologically based pharmacokinetic modeling software tools are being progressively used to forecast the pharmacokinetics and physicochemical properties of bioactive compounds or dosage forms [109]. Therefore, the physicochemical and pharmacokinetic properties of the Calebin-A were predicted using SwissADME, and the results are exhibited in Fig. (**9**). Calebin-A prediction was observed to conform to Lipinski’s rule of five, which showed a total polar surface area (TPSA) equal 102.29 Å2 (Fig. **9**). The bioavailability score was in the range of 55%, suggesting a non-P-glycoprotein (P-gp) substrate and a suitable absorption in the gastrointestinal (GI) tract (Fig. **9**). Calebin-A was reported as a non-inhibitor of cytochrome P450 isoenzyme (CYP1A2, CYP2D6, and CYP2C19) probes.

The conventional way for calculating lipophilicity is the octanol-water partition ratio. Different methods were designed to estimate this point's octanol-water partition ratio (logP). Lipinski's rule applied the Moriguchi-based algorithm to compute logP (MLOGP). The benefits of the Moriguchi technique are the simplicity of programming in all languages without needing a large database of parameter values [110].

According to the calculated ADME parameters, Calebin-A, which is an active compound against NAFLD, could be an oral drug since its MLOGP is equal to 1.49 (Recommended Range:- 2.0 to 6.5) and the log S (Recommended Range: -6.5 to 0.5) is equal to -4.01. Additionally, according to the rule of five, LogP value <5 (ideally 1.35-1.8) is suitable for oral and intestinal absorption, and Calebin-A (Consensus Log Po/w: 2.88) is considered moderately soluble in water [111]. Moreover, the ratio of carbons in the sp3 hybridization is 0.14 for saturation. Although the suitable range of Csp^3^ hybridization was not less than 0.25 [112], due to other ADME properties, Calebin-A could be considered to have sufficient oral bioavailability. Based on ADME findings, Calebin-A indicated good drug-likeness properties without any violations, and synthetic accessibility was equal to 3.24.

## CONCLUSION

Based on computational analysis, our study demonstrated that Calebin-A has 87 common targets with NAFLD. Using PPI network analysis, we represented essential hub genes, including TP53, TNF, STAT3, HSP90AA1, and PTGS2, that are critical in NAFLD and might be influenced by Calebin-A. Here, we reported several pathways, such as those of sphingolipids, VEGFA, C-type lectin receptor, and MAPK, that are important in the progression of NAFLD and could be modulated by Calebin-A. By analyzing Calebin-A interaction with hub proteins, we displayed that Calebin-A could strongly bind to those overexpressed in NAFLD. Consequently, we assumed that Calebin-A might be a promising therapeutic candidate for NAFLD. Further experimental validation, such as *in vitro* and *in vivo* assays, are warranted to confirm the predicted interactions and elucidate the precise mechanisms Calebin-A exerts its anti-steatotic effects. Clinical trials can also be undertaken to evaluate the therapeutic efficacy and safety profile of Calebin-A for potential use in patients with NAFLD.

## Figures and Tables

**Fig. (1):**
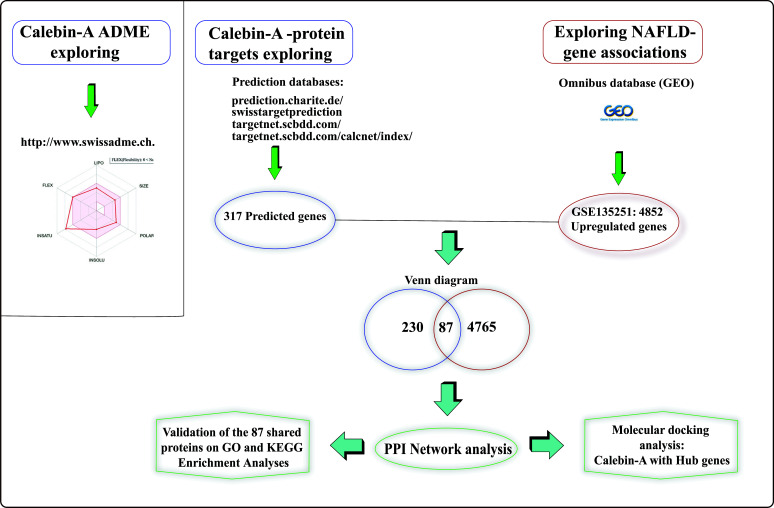
The flow chart of our study.

**Fig. (7):**
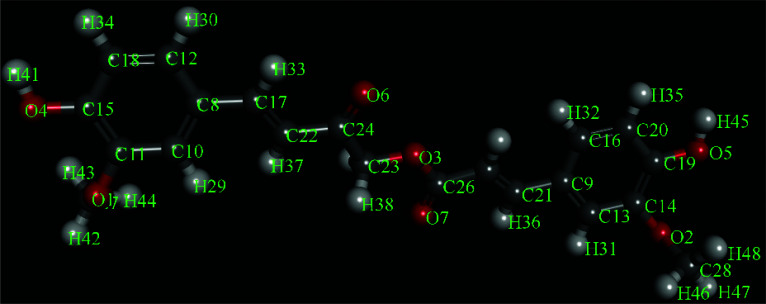
The 3D structure of calebin-A.

**Fig. (8):**
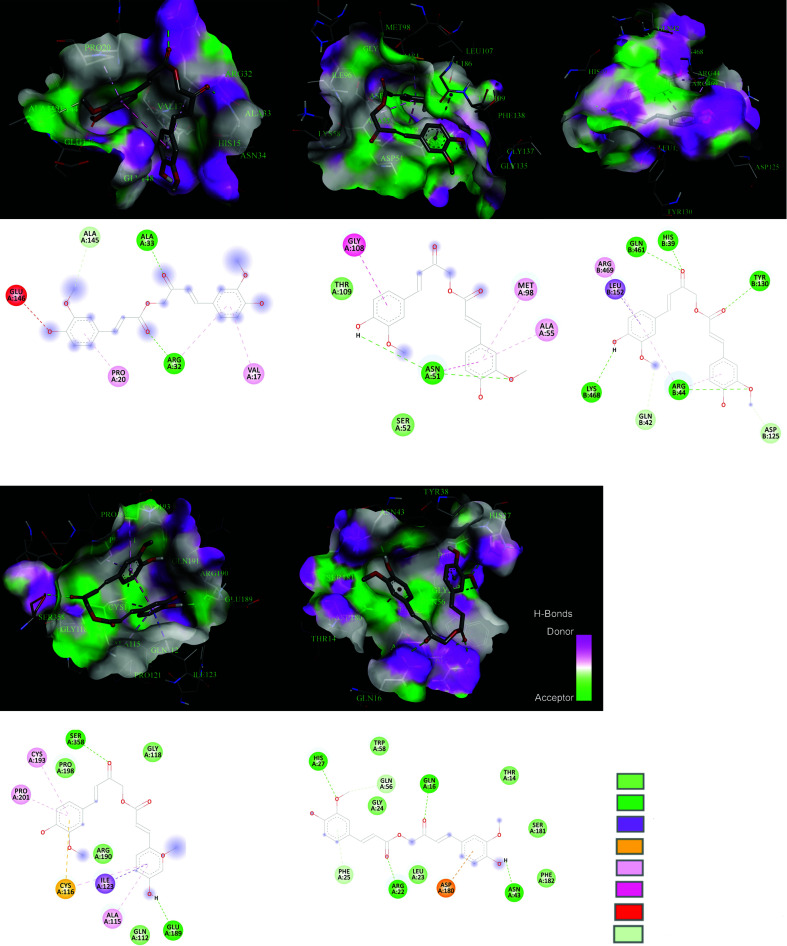
Calebin-A binding site atoms interact with TNF, HSP90AA1, PTGS2, STAT3, and TP53.

**Table 1:** Protein targets of Calebin-A based on several prediction databases.

**Prediction Databases**	**Calebin-A Targets**
swisstargetprediction	100
prediction.charite.de	74
Gdbtools	77
Target net	136
Sum of ALL	317

**Table 2:** The scores of important centralities, fold-change, and FDR (In GSE135251) were reported.

**Gene Symbol**	**Full Name**	**Degree**	**Betweenness**	**Closeness**	**logFC**	**Adjusted-*P*-value (FDR)**
**TP53**	Tumor protein p53	40	0.20	0.63	0.28	4.76E-02
**TNF**	Tumor necrosis factor	37	0.23	0.60	0.91	9.04E-03
**STAT3**	Signal transducer and activator of transcription 3	31	0.11	0.58	0.53	1.93E-03
**HSP90AA1**	Heat shock protein 90 alpha family class A member 1	29	0.11	0.57	0.44	2.37E-02
**PTGS2**	Prostaglandin-endoperoxide synthase 2	23	0.15	0.54	0.58	4.55E-02
**HDAC6**	Histone deacetylase 6	16	0.07	0.48	0.73	3.68E-07
**ABCB1**	ATP-binding cassette subfamily B member 1	14	0.05	0.49	1.54	1.58E-04
**CCT2**	Chaperonin containing TCP1 subunit 2	12	0.06	0.46	0.63	2.54E-02
**NR1I2**	Nuclear receptor subfamily 1 group I member 2	11	0.04	0.47	0.51	1.74E-02
**GUSB**	Glucuronidase beta	7	0.44	0.29	0.24	2.45E-02

**Table 3:** Specifications of the 3D structure proteins selected from the www.rcsb.org database.

**Protein Target**	**PDB ID**	**Method**	**Resolution**	**Positions**
**TNF**	5UUI	X-ray diffraction	1.40 Å	77-233
**STAT3**	6NJS	X-ray diffraction	2.70 Å	127-688
**TP53**	6SL6	X-ray diffraction	1.67 Å	89-311
**PTGS2**	5F19	X-ray diffraction	2.04 Å	2-552
**HSP90AA1**	2YI7	X-ray diffraction	1.40 Å	1-229

**Table 4:** The details of the interaction between calebin-A and five critical hub proteins.

**Calenin-A-protein Interaction**	**Binding Energy (∆G) kcal/mol**	**H-bond**	**Donor Atom**	**Acceptor Atom**	**Distance**
**Calebin A-TNF**	-6.5	A:ARG32:HE - N:UNK1:O	HE	O	2.25859
		A:ALA33:HN - N:UNK1:O	HN	O	2.01347
**Calebin A-HSP90AA1**	-6.9	A:ASN51:HD22 - N:UNK1:O	HD22	O	2.28889
**Calebin A-PTGS2**	-9.3	B:HIS39:HD1 - N:UNK1:O	HD1	O	1.90427
		B:ARG44:HE - N:UNK1:O	HE	O	2.27225
		B:ARG44:HH11 - N:UNK1:O	HH11	O	2.41026
		B:CYS47:HN - N:UNK1:O	HN	O	2.47056
		B:CYS47:HN - N:UNK1:O	HN	O	2.40626
**Calebin A-STAT3**	-7.1	A:GLN112:HE22-N:UNK1:O	HE22	O	2.32814
		A:SER358:HG - N:UNK1:O	HG	O	2.46437
		N:UNK1:H - A:GLU189:O	H	O	1.93606
**Calebin A-TP53**	-6.2	A:ARG22:HE - N:UNK1:O	HE	O	2.06068
		A:ARG22:HH21 - N:UNK1:O	HH21	O	2.42515
		A:PHE25:HN - N:UNK1:O	HN	O	2.47878
		A:PHE25:HN - N:UNK1:O	HN	O	2.43234
		A:TYR38:HH - N:UNK1:O	HH	O	2.27153
		N:UNK1:H - A:ASN43:OD1	H	OD1	1.69958

**Abbreviations:** UNK: Calebin-A, GLY: Glycine, VAL: Valine, LEU: Leucine, PHE: Phenylalanine, SER: Serine, ARG: Arginine, GLU: Glutamic acid, LYS: Lysine, CYS: Cysteine, ALA: Alanine, ASP: Aspartic acid, TYR: Tyrosine, ASN: Asparagine, THR: Threonine, GLN: Glutamine, HIS: Histidine, H: Hydrogen, O: Oxygen, N: Nitrogen, HD1: proton on the delta-Nitrogen of the Histidine.

## Data Availability

The datasets generated and/or analyzed during the current study are not publicly available but are available from the corresponding author at a reasonable request.

## LIST OF ABBREVIATIONS

ABCB1: ATP-binding Cassette Subfamily B Member 1
ADME: Absorption, Distribution, Metabolism, and Excretion
AMPK: AMP-activated Protein Kinase
CCT2: Chaperonin Containing TCP1 Subunit 2
COX-2: Cyclooxygenase Two
DEGs: Differentially Expressed Genes
FAS: Fatty Acid Synthase
GI: Gastrointestinal
GUSB: Glucuronidase Beta
HCC: Hepatocellular Carcinoma
HDAC6: Histone Deacetylase 6
HFD: High-fat Diet
HSC: Hepatic Stellate Cell
HSP90AA1: Heat Shock Protein 90 Alpha Family Class A Member 1
JAKs: Janus Kinases
MAPK: Mitogen-activated Protein Kinase
MCODE: Molecular Complex Detection
NAFLD: Non-alcoholic Fatty Liver Disease
NASH: Non-alcoholic Steatohepatitis
NF-κB: Nuclear Factor Kappa B
NMR: Nuclear Magnetic Resonance
NR1I2: Nuclear Receptor Subfamily 1
Nrf2: Nuclear Factor E2-related Factor 2
P-gp: P-glycoprotein
PPARγ: Peroxisome Proliferator-activated Receptor γ
PPI: Protein-protein Interaction
PTGS2: Prostaglandin-endoperoxide Synthase 2
ROS: Reactive Oxygen Species
SQLE: Squalene Epoxidase
STAT3: Signal Transducer and Activator of Transcription 3
TNF: Tumor Necrosis Factor
TP53: Tumor Protein p53
TPSA: Total Polar Surface Area
VEGFA: Vascular Endothelial Growth Factor A

## References

[1] Li B, Zhang C, Zhan YT (2018). Nonalcoholic fatty liver disease cirrhosis: A review of its epidemiology, risk factors, clinical presentation, diagnosis, management, and prognosis.. Can J Gastroenterol Hepatol.

[2] Machado M.V., Diehl A.M. (2016). Pathogenesis of nonalcoholic steatohepatitis.. Gastroenterology.

[3] Mahmoudi A., Butler A.E., Jamialahmadi T., Sahebkar A. (2022). The role of exosomal miRNA in nonalcoholic fatty liver disease.. J. Cell. Physiol.

[4] Younossi Z., Anstee Q.M., Marietti M., Hardy T., Henry L., Eslam M., George J., Bugianesi E. (2018). Global burden of NAFLD and NASH: Trends, predictions, risk factors and prevention.. Nat. Rev. Gastroenterol. Hepatol..

[5] Wree A, Broderick L, Canbay A, Hoffman HM, Feldstein AE, Eslam M., George J. (2013). From NAFLD to NASH to cirrhosis-new insights into disease mechanisms.. Nat Rev Gastroenterol Hepatol.

[6] Pouwels S., Sakran N., Graham Y., Leal A., Pintar T., Yang W., Kassir R., Singhal R., Mahawar K., Ramnarain D. (2022). Non-alcoholic fatty liver disease (NAFLD): A review of pathophysiology, clinical management and effects of weight loss.. BMC Endocr. Disord..

[7] Kim D.S.H.L., Kim J.Y. (2001). Total synthesis of calebin-A, preparation of its analogues, and their neuronal cell protectivity against β-amyloid insult.. Bioorg. Med. Chem. Lett..

[8] Majeed A., Majeed M., Thajuddin N., Arumugam S., Ali F., Beede K., Adams S.J., Gnanamani M. (2019). Bioconversion of curcumin into calebin-A by the endophytic fungus *Ovatospora brasiliensis* EPE-10 MTCC 25236 associated with *Curcuma caesia*.. AMB Express.

[9] Arafa H.M.M., Hemeida R.A., El-Bahrawy A.I.M., Hamada F.M.A. (2009). Prophylactic role of curcumin in dextran sulfate sodium (DSS)-induced ulcerative colitis murine model.. Food Chem. Toxicol..

[10] Oliveira A.L.D.P., Martinez S.E., Nagabushnam K., Majeed M., Alrushaid S., Sayre C.L., Davies N.M. (2015). Calebin A: Analytical development for pharmacokinetics study, elucidation of pharmacological activities and content analysis of natural health products.. J. Pharm. Pharm. Sci..

[11] Cheng A.L., Hsu C.H., Lin J.K., Hsu M.M., Ho Y.F., Shen T.S., Ko J.Y., Lin J.T., Lin B.R., Ming-Shiang W., Yu H.S., Jee S.H., Chen G.S., Chen T.M., Chen C.A., Lai M.K., Pu Y.S., Pan M.H., Wang Y.J., Tsai C.C., Hsieh C.Y. (2001). Phase I clinical trial of curcumin, a chemopreventive agent, in patients with high-risk or pre-malignant lesions.. Anticancer Res..

[12] Nair A., Amalraj A., Jacob J., Kunnumakkara A.B., Gopi S. (2019). Non-curcuminoids from turmeric and their potential in cancer therapy and anticancer drug delivery formulations.. Biomolecules.

[13] Mahmoudi A., Kesharwani P., Majeed M., Teng Y., Sahebkar A. (2022). Recent advances in nanogold as a promising nanocarrier for curcumin delivery.. Colloids Surf. B Biointerfaces.

[14] Majeed M., Nagabhushanam K., Natarajan S., Bani S., Pandey A., Karri S.K. (2015). Investigation of repeated dose (90 day) oral toxicity, reproductive/developmental toxicity and mutagenic potential of ‘Calebin A’.. Toxicol. Rep..

[15] Lai C.S., Liao S.N., Tsai M.L., Kalyanam N., Majeed M., Majeed A., Ho C.T., Pan M.H. (2015). Calebin-A inhibits adipogenesis and hepatic steatosis in high-fat diet-induced obesity *via* activation of AMPK signaling.. Mol. Nutr. Food Res..

[16] Lee P.S., Lu Y.Y., Nagabhushanam K., Ho C.T., Mei H.C., Pan M.H. (2023). Calebin-A prevents HFD-induced obesity in mice by promoting thermogenesis and modulating gut microbiota.. J. Tradit. Complement. Med..

[17] Brockmueller A., Mueller A.L., Kunnumakkara A.B., Aggarwal B.B., Shakibaei M. (2022). Multifunctionality of Calebin A in inflammation, chronic diseases and cancer.. Front. Oncol..

[18] Oulas A., Minadakis G., Zachariou M., Sokratous K., Bourdakou M.M., Spyrou G.M. (2019). Systems Bioinformatics: Increasing precision of computational diagnostics and therapeutics through network-based approaches.. Brief. Bioinform..

[19] Mahmoudi A., Heydari S., Markina Y.V., Barreto G.E., Sahebkar A. (2022). Role of statins in regulating molecular pathways following traumatic brain injury: A system pharmacology study.. Biomed. Pharmacother..

[20] Mahmoudi A., Atkin S.L., Nikiforov N.G., Sahebkar A. (2022). Therapeutic role of curcumin in diabetes: An analysis based on bioinformatic findings.. Nutrients.

[21] Mahmoudi A., Butler A.E., Majeed M., Banach M., Sahebkar A. (2022). Investigation of the effect of curcumin on protein targets in NAFLD using bioinformatic analysis.. Nutrients.

[22] Mao C., Howard T.D., Sullivan D., Fu Z., Yu G., Parker S.J., Will R., Vander Heide R.S., Wang Y., Hixson J., Van Eyk J., Herrington D.M. (2017). Bioinformatic analysis of coronary disease associated SNPs and genes to identify proteins potentially involved in the pathogenesis of atherosclerosis.. J. Proteom. Genom. Res..

[23] Mahmoudi A., Butler A.E., Banach M., Jamialahmadi T., Sahebkar A. (2023). Identification of potent small-molecule PCSK9 inhibitors based on quantitative structure-activity relationship, pharmacophore modeling, and molecular docking procedure.. Curr. Probl. Cardiol..

[24] Priscilla L., Viol Dhea K., Arif Nur Muhammad A., Muhammad Hermawan W., Rasyadan Taufiq P., Ahmad Affan Ali M. (2022). *In Silico* phytochemical compounds screening of *allium sativum* targeting the Mpro of SARS-CoV-2.. Pharmacogn. J..

[25] Nur Sofiatul A., Viol Dhea K., Muhammad Hermawan W., Ahmad Affan Ali M., Rasyadan Taufiq P., Dora Dayu Rahma T. (2022). *In silico* screening of bioactive compounds from *syzygium cumini L.* and *Moringa oleifera L.* Against SARS-CoV-2 *via* tetra inhibitors.. Pharmacogn. J..

[26] Melge A.R., Manzoor K., Nair S.V., Mohan C.G. (2019). *In silico* modeling of FDA-approved drugs for discovery of anti-cancer agents: A drug-repurposing approach. *In silico* drug design..

[27] Mahmoudi A., Atkin S.L., Jamialahmadi T., Banach M., Sahebkar A. (2022). Effect of curcumin on attenuation of liver cirrhosis *via* genes/proteins and pathways: A system pharmacology study.. Nutrients.

[28] Daina A., Michielin O., Zoete V. (2019). Swiss target prediction: Updated data and new features for efficient prediction of protein targets of small molecules.. Nucleic Acids Res..

[29] Yao Z.J., Dong J., Che Y.J., Zhu M.F., Wen M., Wang N.N., Wang S., Lu A.P., Cao D.S. (2016). TargetNet: A web service for predicting potential drug–target interaction profiling *via* multi-target SAR models.. J. Comput. Aided Mol. Des..

[30] Gallo K., Goede A., Preissner R., Gohlke B.O. (2022). SuperPred 3.0: Drug classification and target prediction—a machine learning approach.. Nucleic Acids Res..

[31] Awale M., Reymond J.L. (2017). The polypharmacology browser: A web-based multi-fingerprint target prediction tool using ChEMBL bioactivity data.. J. Cheminform..

[32] Clough E., Barrett T. (2016). The gene expression omnibus database.. Methods Mol. Biol..

[33] Szklarczyk D., Gable A.L., Nastou K.C., Lyon D., Kirsch R., Pyysalo S., Doncheva N.T., Legeay M., Fang T., Bork P., Jensen L.J., von Mering C. (2021). The STRING database in 2021: Customizable protein–protein networks, and functional characterization of user-uploaded gene/measurement sets.. Nucleic Acids Res..

[34] Majeed A., Mukhtar S. (2023). Protein–protein interaction network exploration using cytoscape.. Methods Mol. Biol..

[35] Bader G.D., Hogue C.W.V. (2003). An automated method for finding molecular complexes in large protein interaction networks.. BMC Bioinformatics.

[36] Kim S., Chen J., Cheng T., Gindulyte A., He J., He S., Li Q., Shoemaker B.A., Thiessen P.A., Yu B., Zaslavsky L., Zhang J., Bolton E.E. (2021). PubChem in 2021: New data content and improved web interfaces.. Nucleic Acids Res..

[37] Eberhardt J., Santos-Martins D., Tillack A.F., Forli S. (2021). AutoDock vina 1.2.0: New docking methods, expanded force field, and python bindings.. J. Chem. Inf. Model..

[38] Sharma P.K., Yadav I.S. (2022). Biological databases and their application. Bioinformatics..

[39] Pettersen E.F., Goddard T.D., Huang C.C., Couch G.S., Greenblatt D.M., Meng E.C., Ferrin T.E. (2004). UCSF Chimera—A visualization system for exploratory research and analysis.. J. Comput. Chem..

[40] Dallakyan S., Olson A.J. (2015). Small-molecule library screening by docking with PyRx. Chemical biology..

[41] Bindea G., Mlecnik B., Hackl H., Charoentong P., Tosolini M., Kirilovsky A., Fridman W.H., Pagès F., Trajanoski Z., Galon J. (2009). ClueGO: A Cytoscape plug-in to decipher functionally grouped gene ontology and pathway annotation networks.. Bioinformatics.

[42] Qiu Y-Q., Dubitzky W., Wolkenhauer O., Cho K-H., Yokota H. (2013). KEGG pathway database.. Encyclopedia of Systems Biology..

[43] Panahi Y, Sahebkar A., Amiri M, Davoudi SM, Beiraghdar F, Hoseininejad SL, Kolivand M. (2012). Improvement of sulphur mustard-induced chronic pruritus, quality of life and antioxidant status by curcumin: results of a randomised, double-blind, placebo-controlled trial.. Br J Nutr..

[44] Cicero A.F.G., Sahebkar A., Fogacci F., Bove M., Giovannini M., Borghi C. (2020). Effects of phytosomal curcumin on anthropometric parameters, insulin resistance, cortisolemia and non-alcoholic fatty liver disease indices: A double-blind, placebo-controlled clinical trial.. Eur. J. Nutr..

[45] Kahkhaie K.R., Mirhosseini A., Aliabadi A., Mohammadi A., Mousavi M.J., Haftcheshmeh S.M., Sathyapalan T., Sahebkar A. (2019). Curcumin: A modulator of inflammatory signaling pathways in the immune system.. Inflammopharmacology.

[46] Keihanian F., Saeidinia A., Bagheri R.K., Johnston T.P., Sahebkar A. (2018). Curcumin, hemostasis, thrombosis, and coagulation.. J. Cell. Physiol..

[47] Khayatan D., Razavi S.M., Arab Z.N., Niknejad A.H., Nouri K., Momtaz S., Gumpricht E., Jamialahmadi T., Abdolghaffari A.H., Barreto G.E., Sahebkar A. (2022). Protective effects of curcumin against traumatic brain injury.. Biomed. Pharmacother..

[48] Marjaneh R.M., Rahmani F., Hassanian S.M., Rezaei N., Hashemzehi M., Bahrami A., Ariakia F., Fiuji H., Sahebkar A., Avan A., Khazaei M. (2018). Phytosomal curcumin inhibits tumor growth in colitis‐associated colorectal cancer.. J. Cell. Physiol..

[49] Mohajeri M., Sahebkar A. (2018). Protective effects of curcumin against doxorubicin-induced toxicity and resistance: A review.. Crit. Rev. Oncol. Hematol..

[50] Mohammadi A., Blesso C.N., Barreto G.E., Banach M., Majeed M., Sahebkar A. (2019). Macrophage plasticity, polarization and function in response to curcumin, a diet-derived polyphenol, as an immunomodulatory agent.. J. Nutr. Biochem..

[51] Mokhtari-Zaer A., Marefati N., Atkin S.L., Butler A.E., Sahebkar A. (2019). The protective role of curcumin in myocardial ischemia–reperfusion injury.. J. Cell. Physiol..

[52] Panahi Y., Fazlolahzadeh O., Atkin S.L., Majeed M., Butler A.E., Johnston T.P., Sahebkar A. (2019). Evidence of curcumin and curcumin analogue effects in skin diseases: A narrative review.. J. Cell. Physiol..

[53] Rodriguez-Cuenca S., Pellegrinelli V., Campbell M., Oresic M., Vidal-Puig A. (2017). Sphingolipids and glycerophospholipids: The “ying and yang” of lipotoxicity in metabolic diseases.. Prog. Lipid Res..

[54] Musso G., Cassader M., Paschetta E., Gambino R. (2018). Bioactive lipid species and metabolic pathways in progression and resolution of nonalcoholic steatohepatitis.. Gastroenterology.

[55] Sztolsztener K., Konstantynowicz-Nowicka K., Harasim-Symbor E., Chabowski A. (2021). Time-dependent changes in hepatic sphingolipid accumulation and PI3K/Akt/mTOR signaling pathway in a rat model of NAFLD.. Int. J. Mol. Sci..

[56] Shen H., Yu H., Li Q., Wei Y., Fu J., Dong H., Cao D., Guo L., Chen L., Yang Y., Xu Y., Wu M., Wang H., Chen Y. (2022). Hepatocyte-derived VEGFA accelerates the progression of non-alcoholic fatty liver disease to hepatocellular carcinoma *via* activating hepatic stellate cells.. Acta Pharmacol. Sin..

[57] Peluso I., Yarla N.S., Ambra R., Pastore G., Perry G. (2019). MAPK signalling pathway in cancers: Olive products as cancer preventive and therapeutic agents.. Semin. Cancer Biol..

[58] Lawan A., Bennett A.M. (2017). Mitogen-activated protein kinase regulation in hepatic metabolism.. Trends Endocrinol. Metab..

[59] Wu L., Liu Y., Zhao Y., Li M., Guo L. (2020). Targeting DUSP7 signaling alleviates hepatic steatosis, inflammation and oxidative stress in high fat diet (HFD)-fed mice *via* suppression of TAK1.. Free Radic. Biol. Med..

[60] Zai W., Chen W., Wu Z., Jin X., Fan J., Zhang X., Luan J., Tang S., Mei X., Hao Q., Liu H., Ju D. (2019). Targeted interleukin-22 gene delivery in the liver by polymetformin and penetratin-based hybrid nanoparticles to treat nonalcoholic fatty liver disease.. ACS Appl. Mater. Interfaces.

[61] Zhang L., Tian R., Yao X., Zhang X.J., Zhang P., Huang Y., She Z.G., Li H., Ji Y.X., Cai J. (2021). Milk fat globule–epidermal growth factor–factor 8 improves hepatic steatosis and inflammation.. Hepatology.

[62] Lu Y., Jiang Z., Dai H., Miao R., Shu J., Gu H., Liu X., Huang Z., Yang G., Chen A.F., Yuan H., Li Y., Cai J. (2018). Hepatic leukocyte immunoglobulin‐like receptor B4 (LILRB4) attenuates nonalcoholic fatty liver disease *via* SHP1‐TRAF6 pathway.. Hepatology.

[63] Holbrook J., Lara-Reyna S., Jarosz-Griffiths H., McDermott M.F. (2019). Tumour necrosis factor signalling in health and disease.. F1000 Res..

[64] Jang D., Lee A.H., Shin H.Y., Song H.R., Park J.H., Kang T.B., Lee S.R., Yang S.H. (2021). The role of tumor necrosis factor alpha (TNF-α) in autoimmune disease and current TNF-α inhibitors in therapeutics.. Int. J. Mol. Sci..

[65] Lu S., Wang Y., Liu J. (2022). Tumor necrosis factor-α signaling in nonalcoholic steatohepatitis and targeted therapies.. J. Genet. Genomics.

[66] Wandrer F., Liebig S., Marhenke S., Vogel A., John K., Manns M.P., Teufel A., Itzel T., Longerich T., Maier O., Fischer R., Kontermann R.E., Pfizenmaier K., Schulze-Osthoff K., Bantel H. (2020). TNF-Receptor-1 inhibition reduces liver steatosis, hepatocellular injury and fibrosis in NAFLD mice.. Cell Death Dis..

[67] Anderson N., Borlak J. (2008). Molecular mechanisms and therapeutic targets in steatosis and steatohepatitis.. Pharmacol. Rev..

[68] Cobbina E., Akhlaghi F. (2017). Non-alcoholic fatty liver disease (NAFLD): Pathogenesis, classification, and effect on drug metabolizing enzymes and transporters.. Drug Metab. Rev..

[69] Ferreira A.V.M., Mario É.G., Porto L.C.J., Andrade S.P., Botion L.M. (2011). High-carbohydrate diet selectively induces tumor necrosis factor-α production in mice liver.. Inflammation.

[70] Oliveira M.C., Menezes-Garcia Z., Arifa R.D.N., Paula T.P., Andrade J.M.O., Santos S.H.S., Menezes G.B., Souza D.G., Teixeira M.M., Ferreira A.V.M. (2015). Platelet-activating factor modulates fat storage in the liver induced by a high-refined carbohydrate-containing diet.. J. Nutr. Biochem..

[71] Saadati S., Sadeghi A., Mansour A., Yari Z., Poustchi H., Hedayati M., Hatami B., Hekmatdoost A. (2019). Curcumin and inflammation in non-alcoholic fatty liver disease: A randomized, placebo controlled clinical trial.. BMC Gastroenterol..

[72] Hui J.M., Hodge A., Farrell G.C., Kench J.G., Kriketos A., George J. (2004). Beyond insulin resistance in NASH: TNF-? or adiponectin?. Hepatology.

[73] Wellen K.E., Hotamisligil G.S. (2005). Inflammation, stress, and diabetes.. J. Clin. Invest..

[74] Uysal K.T., Wiesbrock S.M., Marino M.W., Hotamisligil G.S. (1997). Protection from obesity-induced insulin resistance in mice lacking TNF-α function.. Nature.

[75] Crespo J., Cayón A., Fernández-Gil P., Hernández-Guerra M., Mayorga M., Domínguez-Díez A., Fernández-Escalante J.C., Pons-Romero F. (2001). Gene expression of tumor necrosis factor [alpha ] and TNF-receptors, p55 and p75, in nonalcoholic steatohepatitis patients.. Hepatology.

[76] Tomita K., Tamiya G., Ando S., Ohsumi K., Chiyo T., Mizutani A., Kitamura N., Toda K., Kaneko T., Horie Y., Han J.Y., Kato S., Shimoda M., Oike Y., Tomizawa M., Makino S., Ohkura T., Saito H., Kumagai N., Nagata H., Ishii H., Hibi T. (2006). Tumour necrosis factor signalling through activation of Kupffer cells plays an essential role in liver fibrosis of non-alcoholic steatohepatitis in mice.. Gut.

[77] Tyagi A.K., Prasad S., Majeed M., Aggarwal B.B. (2017). Calebin A, a novel component of turmeric, suppresses NF-κB regulated cell survival and inflammatory gene products leading to inhibition of cell growth and chemosensitization.. Phytomedicine.

[78] Buhrmann C, Kunnumakkara AB, Popper B, Majeed M, Aggarwal BB, Shakibaei M (2020). Calebin a potentiates the effect of 5-FU and TNF-beta *(Lymphotoxin alpha)* against human colorectal cancer cells: Potential role of NF-kappa B.. Inter. J. Mole. Sci..

[79] Buhrmann C., Popper B., Kunnumakkara A.B., Aggarwal B.B., Shakibaei M. (2019). Evidence that calebin a, a component of curcuma longa suppresses NF-κB mediated proliferation, invasion and metastasis of human colorectal cancer induced by TNF-β *(Lymphotoxin)*.. Nutrients.

[80] Buhrmann C., Kunnumakkara A.B., Kumar A., Samec M., Kubatka P., Aggarwal B.B., Shakibaei M. (2021). Multitargeting effects of calebin A on malignancy of CRC cells in multicellular tumor microenvironment.. Front. Oncol..

[81] Mueller A.L., Brockmueller A., Kunnumakkara A.B., Shakibaei M. (2022). Calebin A, a compound of turmeric, down-regulates inflammation in tenocytes by NF-κB/Scleraxis signaling.. Int. J. Mol. Sci..

[82] Zhao J., Qi Y.F., Yu Y.R. (2021). STAT3: A key regulator in liver fibrosis.. Ann. Hepatol..

[83] Stärkel P., De Saeger C., Leclercq I., Strain A., Horsmans Y. (2005). Deficient Stat3 DNA-binding is associated with high Pias3 expression and a positive anti-apoptotic balance in human end-stage alcoholic and hepatitis C cirrhosis.. J. Hepatol..

[84] Stärkel P., Bishop K., Horsmans Y., Strain A.J. (2003). Expression and DNA-binding activity of signal transducer and activator of transcription 3 in alcoholic cirrhosis compared to normal liver and primary biliary cirrhosis in humans.. Am. J. Pathol..

[85] Choi S., Jung H.J., Kim M.W., Kang J.H., Shin D., Jang Y.S., Yoon Y.S., Oh S.H. (2019). A novel STAT3 inhibitor, STX-0119, attenuates liver fibrosis by inactivating hepatic stellate cells in mice.. Biochem. Biophys. Res. Commun..

[86] Younes M., Zhang L., Fekry B., Eckel-Mahan K. (2022). Expression of p-STAT3 and c-Myc correlates with P2-HNF4α expression in nonalcoholic fatty liver disease (NAFLD).. Oncotarget.

[87] Park J., Zhao Y., Zhang F., Zhang S., Kwong A.C., Zhang Y., Hoffmann H.H., Bushweller L., Wu X., Ashbrook A.W., Stefanovic B., Chen S., Branch A.D., Mason C.E., Jung J.U., Rice C.M., Wu X. (2023). IL-6/STAT3 axis dictates the PNPLA3-mediated susceptibility to non-alcoholic fatty liver disease.. J. Hepatol..

[88] Liu Y., Wang X., Zeng S., Zhang X., Zhao J., Zhang X. (2018). The natural polyphenol curcumin induces apoptosis by suppressing STAT3 signaling in esophageal squamous cell carcinoma 06 biological sciences 0601 biochemistry and cell biology 11 medical and health sciences 1112 oncology and carcinogenesis.. J. Exp. Clin. Cancer Res..

[89] Liu L., Liu Y.L., Liu G.X., Chen X., Yang K., Yang Y.X., Xie Q., Gan H.K., Huang X.L., Gan H.T. (2013). Curcumin ameliorates dextran sulfate sodium-induced experimental colitis by blocking STAT3 signaling pathway.. Int. Immunopharmacol..

[90] Mahata S., Behera S.K., Kumar S., Sahoo P.K., Sarkar S., Fazil M.H.U.T., Nasare V.D. (2022). *In-silico* and *in-vitro* investigation of STAT3-PIM1 heterodimeric complex: Its mechanism and inhibition by curcumin for cancer therapeutics.. Int. J. Biol. Macromol..

[91] Hahn Y.I., Kim S.J., Choi B.Y., Cho K.C., Bandu R., Kim K.P., Kim D.H., Kim W., Park J.S., Han B.W., Lee J., Na H.K., Cha Y.N., Surh Y.J. (2018). Curcumin interacts directly with the Cysteine 259 residue of STAT3 and induces apoptosis in H-Ras transformed human mammary epithelial cells.. Sci. Rep..

[92] Chakraborty A., Uechi T., Kenmochi N. (2011). Guarding the ‘translation apparatus’: Defective ribosome biogenesis and the p53 signaling pathway.. Wiley Interdiscip. Rev. RNA.

[93] Pani G., Fusco S., Colavitti R., Borrello S., Maggiano N., Cravero A.A.M., Farré S.M., Galeotti T., Koch O.R. (2004). Abrogation of hepatocyte apoptosis and early appearance of liver dysplasia in ethanol-fed p53-deficient mice.. Biochem. Biophys. Res. Commun..

[94] Derdak Z., Lang C.H., Villegas K.A., Tong M., Mark N.M., de la Monte S.M., Wands J.R. (2011). Activation of p53 enhances apoptosis and insulin resistance in a rat model of alcoholic liver disease.. J. Hepatol..

[95] Derdak Z., Villegas K.A., Harb R., Wu A.M., Sousa A., Wands J.R. (2013). Inhibition of p53 attenuates steatosis and liver injury in a mouse model of non-alcoholic fatty liver disease.. J. Hepatol..

[96] Farrell G.C., Larter C.Z., Hou J.Y., Zhang R.H., Yeh M.M., Williams J., Dela Peňa A., Francisco R., Osvath S.R., Brooling J., Teoh N., Sedger L.M. (2009). Apoptosis in experimental NASH is associated with p53 activation and TRAIL receptor expression.. J. Gastroenterol. Hepatol..

[97] Panasiuk A., Dzieciol J., Panasiuk B., Prokopowicz D. (2006). Expression of p53, Bax and Bcl-2 proteins in hepatocytes in non-alcoholic fatty liver disease.. World J. Gastroenterol..

[98] Yahagi N., Shimano H., Matsuzaka T., Sekiya M., Najima Y., Okazaki S., Okazaki H., Tamura Y., Iizuka Y., Inoue N., Nakagawa Y., Takeuchi Y., Ohashi K., Harada K., Gotoda T., Nagai R., Kadowaki T., Ishibashi S., Osuga J., Yamada N. (2004). p53 involvement in the pathogenesis of fatty liver disease.. J. Biol. Chem..

[99] Sun H., Li L., Li W., Yang F., Zhang Z., Liu Z., Du W. (2021). p53 transcriptionally regulates SQLE to repress cholesterol synthesis and tumor growth.. EMBO Rep..

[100] Liou W.S., Lin C., Lee P.S., Kalyanam N., Ho C.T., Pan M.H. (2016). Calebin-A induces cell cycle arrest in human colon cancer cells and xenografts in nude mice.. J. Funct. Foods.

[101] Xie Y., Chen L., Xu Z., Li C., Ni Y., Hou M., Chen L., Chang H., Yang Y., Wang H., He R., Chen R., Qian L., Luo Y., Zhang Y., Li N., Zhu Y., Ji M., Liu Y. (2021). Predictive modeling of MAFLD based on Hsp90α and the therapeutic application of teprenone in a diet-induced mouse model.. Front. Endocrinol..

[102] Asadzadeh-Aghdaei H., Zadeh-Esmaeel M.M., Esmaeili S., Rezaei Tavirani M., Rezaei Tavirani S., Mansouri V., Montazer F. (2019). Effects of high fat medium conditions on cellular gene expression profile: A network analysis approach.. Gastroenterol. Hepatol. Bed Bench.

[103] Cai Y., Jogasuria A., Yin H., Xu M.J., Hu X., Wang J., Kim C., Wu J., Lee K., Gao B., You M. (2016). The detrimental role played by lipocalin-2 in alcoholic fatty liver in mice.. Am. J. Pathol..

[104] Lv Y., Gong L., Wang Z., Han F., Liu H., Lu X., Liu L. (2015). Curcumin inhibits human cytomegalovirus by downregulating heat shock protein 90.. Mol. Med. Rep..

[105] Chan P.C., Liao M.T., Hsieh P.S. (2019). The dualistic effect of COX-2-mediated signaling in obesity and insulin resistance.. Int. J. Mol. Sci..

[106] Liu Y., Liu X., Zhou W., Zhang J., Wu J., Guo S., Jia S., Wang H., Li J., Tan Y. (2022). Integrated bioinformatics analysis reveals potential mechanisms associated with intestinal flora intervention in nonalcoholic fatty liver disease.. Medicine.

[107] Novaes J., Lillico R., Sayre C., Nagabushanam K., Majeed M., Chen Y., Ho E., Oliveira A., Martinez S., Alrushaid S., Davies N., Lakowski T. (2017). Disposition, metabolism and histone deacetylase and acetyltransferase inhibition activity of tetrahydrocurcumin and other curcuminoids.. Pharmaceutics.

[108] Seibert K., Masferrer J.L. (1994). Role of inducible cyclooxygenase (COX-2) in inflammation.. Receptor.

[109] Palanichamy C., Pavadai P., Panneerselvam T., Arunachalam S., Babkiewicz E., Ram Kumar Pandian S., Shanmugampillai Jeyarajaguru K., Nayak Ammunje D., Kannan S., Chandrasekaran J., Sundar K., Maszczyk P., Kunjiappan S. (2022). Aphrodisiac performance of bioactive compounds from *mimosa pudica* linn.: *In silico* molecular docking and dynamics simulation approach.. Molecules.

[110] Krstulović L., Leventić M., Rastija V., Starčević K., Jirouš M., Janić I., Karnaš M., Lasić K., Bajić M., Glavaš-Obrovac L. (2023). Novel 7-chloro-4-aminoquinoline-benzimidazole hybrids as inhibitors of cancer cells growth: Synthesis, antiproliferative activity, *in silico* adme predictions, and docking.. Molecules.

[111] Bhal S.K. (2007). LogP—making sense of the value..

[112] Daina A., Michielin O., Zoete V. (2017). SwissADME: A free web tool to evaluate pharmacokinetics, drug-likeness and medicinal chemistry friendliness of small molecules.. Sci. Rep..

